# Special Issue “Advances in Thermal and Mechanical Properties of Polymeric Materials”

**DOI:** 10.3390/ma17010079

**Published:** 2023-12-23

**Authors:** Agnieszka Kijo-Kleczkowska, Adam Gnatowski

**Affiliations:** Faculty of Mechanical Engineering and Computer Science, Czestochowa University of Technology, Dabrowskiego 69, 42-201 Czestochowa, Poland

The Special Issue “Advances in Thermal and Mechanical Properties of Polymeric Materials” aimed to publish papers that deal with the thermomechanical and electrical properties of polymers and their composites with other materials. It is important to recognize the applicability of various fillers in polymer composites in order to create new composites and modify existing composites. Advancements in the engineering of polymeric materials, including the search for innovative polymer composites with specific properties, resulted in the expansion of their application, especially in automotive, construction, energy, packaging, and medical industries. Practical application of new polymeric materials requires knowledge of their mechanical, electrical, and thermal properties, as well as the recognition of changes in these properties during the operation and destruction of polymers. The environmental aspect of research is important, including the combustion/co-combustion of polymers, the thermal use of polymer waste with energy recovery, as well as other uses of recycled polymer materials. It is important to conduct model studies on changes in the properties of polymeric materials and the computer simulation of the exploitation and thermal processes of polymers.

Starting 20 December 2021, this Special Issue has attracted the interest of respected researchers from all over the world. The success of this issue is evidenced by the publication of 12 papers that underwent a rigorous review process conducted by experts in the field. Guest Editors congratulate all the authors of the published works and thank the reviewers for their time, very valuable comments and suggestions that raised the rank and substantive value of our Special Issue. The success of this Special Issue would not be possible without the constant contact and kind support of the Section Managing Editor, Ms. Fay Liu, who should be gratefully thanked for her dedication and commitment. Guest Editors also thank the Editors-in-Chief of *Materials* for the opportunity to collaborate on the journal and congratulate them on their stewardship of a globally respected journal.

The diligence, creativity, friendly and dynamic cooperation of all those mentioned above contributed to the success of this Special Issue.

Below, a brief review of the papers published in the collection of this Special Issue is presented.

In paper [1], the authors presented deals with the aspect of the utilization of waste polyethylene (HDPE) as a matrix in composites with filler in the form of cement at 5 and 10%. Comparative thermomechanical (DSC, tensile strength, DMTA), microstructure and flammability results were presented for HDPE samples and their composites with cement. It was found that the addition of cement as a filler to polyethylene made it possible to obtain composites with good thermomechanical properties.

Bragov et al. [2] emphasized that comfort is an important quality criterion, especially for sportswear. The authors offered a statistically significant model of multiple linear regression equations to predict the thermal comfort of knitted fabric. They showed, among other aspects, that yarn’s linear density, yarn short fiber hairiness, and mass per unit area of knitted fabric has the greatest impact on heat resistance.

The authors of paper [3] stated that water temperature affects the peak pressure damping of transient flows in viscoelastic pipes. In the paper, the Kelvin–Voigt model with both a quasi-steady friction model and a modified Brunone model was employed. Based on experimental data, the accuracy of simulated peak pressure damping was verified at four different water temperatures. Numerical results indicated that the viscoelastic properties of pipes have a greater impact on peak pressure damping than their frictional properties at 25, 31, and 38.5 °C.

Article [4] discussed the influence of pressure and temperature on material density and thermal conductivity of biomass compacted into briquette samples. The pressure of 200 bar was concluded to be the most economically viable in briquetting technology in the tests conducted. The average thermal conductivity for the compacted biomass was determined at a value of 0.048 ± 0.001 W/(K∙m). The methodology described in the paper for thermal conductivity determination was found to be a reliable tool; therefore, it can be proposed for other applications.

In paper [5], a series of novel luminescent hybrid materials was presented, designed based on the top–down procedure. The resulting materials were characterized using Fourier transform infrared spectroscopy and thermal analysis methods, as well as luminescence spectroscopy, and the homogeneity of the resulting materials was investigated by means of optical microscopy. The authors stated, among other aspects, that all obtained materials exhibited good thermal stability in both oxidizing and inert atmospheres.

The aim of paper [6] was the study of the behavior against water of four new plaster-based composite materials using additions of two types of super-absorbent polymers (sodium polyacrylate and potassium polyacrylate) and a lightening material (vermiculite) in their manufacturing process. The transmission of water vapor through the samples and the water absorption capacity of the samples were studied, too. The results of this study showed that composites with the addition of super-absorbent polymers as well as vermiculite significantly improve their water performance compared to traditional materials up to a 7.3% water absorption with a minimal (13%) reduction in mechanical strength compared to current materials with similar additions.

Paper [7] undertook a study of the effect of storage at various relative humidities on the structure, molecular mobility, and mechanical properties of thermoplastic starch (TPS) and its nanocomposite. The reinforcing effect of TPS-montmorillonite (MMT) nanoparticles manifested itself by improving the mechanical properties of nanocomposite samples when compared to TPS samples and led to lower molecular mobility of TPS components. From the point of view of final material application in practice, the authors concluded that the best performance of the studied TPS-based nanocomposite is reached when stored at relative humidities of 55%.

In paper [8], the authors presented the impact of removing contaminants from post-consumer recycled polypropylene on its degradation and properties by implementing a systematic approach for decontamination by washing. Four lots of recycled plastics with different degrees of contamination were evaluated via Fourier transform infrared, melt flow indexer, and differential scanning calorimetry and tested for tensile strength. The authors concluded that the most promising washing procedure for improving the recycled polypropylene quality is cold washing, as it showed very similar results to washing with hot water and hot washing with cleaning agent methods, with the advantages of energy savings and environmental safety.

Paper [9] introduced novel research into specific mechanical properties of composites produced by 3D printing using Continuous Fiber Fabrication. Nylon was used as the composite base material, while carbon constituted the reinforcement element. The authors found, among other things, that the addition of carbon fiber to the base material was proven to increase the volume of defects in the sample. They observed an increase in the tensile strength of up to 12 times higher compared to the sample without reinforcement and stated that to affect the magnitude of work, the fiber reinforcement needed to break the specimens.

In paper [10], the authors presented the combustion results of selected fuels and waste: hard coal, coal sludge, coke waste, sewage sludge, paper waste, biomass waste and polymer waste. An interesting element of paper was the chemical analysis of the XRF of the materials. The authors provided a comparative analysis of pollutant emissions, especially mercury emission, during combustion, and stated that coke waste and sewage sludge are distinguished by their high mercury content. The value of Hg emission during combustion depends on the initial mercury content in the waste. The results of the conducted tests show the adequacy of mercury release during the considered materials’ combustion in terms of emissions of the other compounds considered (CO, CO_2_, SO_2_, H_2_S and NO_x_). The addition of a 10% considered polymer to coal fuels leads to a reduction in mercury emissions in exhaust gases compared to coal fuels alone. This reduction is 53.72% in the case of hard coal (90%) + polymer waste (10%) and 26.36% in the case of coal sludge (90%) + polymer waste (10%).

The authors of paper [11] stated that multi-material components are difficult or even impossible to recycle. This article assessed the potential of polypropylene injection molding grades to produce parts with varied transparency (haze) in order to obtain, in the future, mono-material products with a property gradient. Several polypropylene grades of different types were identified, and the haze of parts injected under various thermal processing conditions was characterized. The results showed a significant dependence of haze on the tested materials, thermal processing conditions and thicknesses of the samples.

Rodriguez et al. [12] presented the thermal conductivity in different filaments that fused deposition modeling technology uses because there are multiple applications for these additive manufacturing products in the field of thermal insulation. The tests were carried out on a set of seven different materials with 100% fabrication density, from base materials to materials with metal inclusions. This study showed that the parts manufactured with aluminum inclusions have higher thermal conductivity, at 0.40 ± 0.05 W/m·K, compared to other materials with high mechanical and thermal resistance, such as thermoplastic polyurethane, with a conductivity of 0.26 ± 0.05 W/m·K.

Guest Editors congratulate the authors for interesting results presented in their articles and invite all scientists in this field to publish innovative articles in the new Special Issue, “Advances in Thermal and Mechanical Properties of Polymeric Materials (2nd Edition)”.
